# Characterization, kinetic, and isotherm data for adsorption of Pb^2+^ from aqueous solution by adsorbent from mixture of bagasse-bentonite

**DOI:** 10.1016/j.dib.2017.11.098

**Published:** 2017-12-06

**Authors:** Eko Prasetyo Kuncoro, Dwi Ratri Mitha Isnadina, Handoko Darmokoesoemo, Oktiani Rahmanita Fauziah, Heri Septya Kusuma

**Affiliations:** a Department of Biology, Faculty of Science and Technology, Airlangga University, 60115, Indonesia; b Department of Chemistry, Faculty of Science and Technology, Airlangga University, 60115, Indonesia; c Department of Chemical Engineering, Faculty of Industrial Technology, Institut Teknologi Sepuluh Nopember, 60111, Indonesia

**Keywords:** Adsorption, Bagasse, Bentonite, Pb^2+^, Composite adsorbent

## Abstract

The usage of wastes of bagasse would be admirable from environmental and solid waste management point of view. Thus, herein, this data set present a facile method for providing an adsorbent from mixture of bagasse-bentonite. The prepared adsorbent was applied to remove Pb^2+^ from aqueous solution. It was conducted in laboratory scale using completely randomized design with variations in mixed mass ratio (1:0, 1:1, 1:2, 1:3, 2:1, 3:1), pH (2, 3, 4, 5, 6, 7) and contact time (5, 10, 30, 45, 90, 120, 180 min) and the adsorption technique was batch technique. The mixed adsorbent with 3:1 of mass ratio provided the highest Pb^2+^ adsorption efficiency of 97.31%. The optimum pH of Pb^2+^ adsorption was 5 and contact time was efficient at 45 min giving adsorption efficiency of 94.76% and 93.38%. The characterization data of the adsorbent were analyzed using XRF and FTIR methods. The XRF test results showed the changes of elemental content in adsorbent after the adsorption indicated that adsorbent can absorb Pb^2+^. The FTIR test results showed that adsorbent has a functional group that is useful in adsorption process. Adsorption of Pb^2+^ by adsorbent from mixture of bagasse-bentonite follows pseudo second order model with correlation coefficient value of 99.99% (*R*^2^ = 0.9999) and Freundlich isotherm model with correlation coefficient value of 90.05% (*R*^2^ = 0.9005). The acquired data indicated that the adsorption of Pb^2+^ by the adsorbent prepared from mixture of bagasse-bentonite is a promising technique for treating Pb-bearing wastewaters.

**Specifications Table**

**Table:** 

Subject area	*Chemical Engineering*
More specific subject area	*Adsorption*
Type of data	*Table, image, figure*
How data was acquired	– *The uptake of Pb*^*2+*^ *by the adsorbent (q*_*e*_*) was determined based on the subtraction of the initial and final concentration of adsorbate* – *Fourier transform infrared (FTIR) spectroscopy (Shimadzu, IRPrestige 21), X-ray fluorescence (PANalytical, Minipal 4) was used for determine the characteristics of the adsorbent* – *The Pb*^*2+*^ *concentration measurement was performed by Atomic Absorption Spectrophotometer (Shimadzu, AA-7000)*
Data format	*Analyzed*
Experimental factors	– *The adsorbent of bagasse-bentonite was prepared from mixture of bagasse and bentonite that have been weighed in accordance with the ratio of 3:1* – *The adsorbent of bagasse-bentonite was heated in the oven at 105 *^*o*^*C for 24 h until the mixture is already dry* – *Data of bagasse-bentonite were acquired for Pb*^*2+*^ *removal from aqueous solution*
Experimental features	*The adsorbent of bagasse-bentonite for Pb*^*2+*^*adsorption from aqueous solution*
Data source location	*Airlangga University, Surabaya, Indonesia*
Data accessibility	*Data are accessible with the article*

**Value of the data**

• The newly synthesized adsorbent has a good potential application in related of wastewater treatment
• The isotherm data will be informative and useful for predicting and modeling the adsorption capacity and mechanism of lead removal by the adsorbent
• The acquired data will be advantageous for the scientific community wanting to scale up and design an adsorption column with adsorbent of bagasse-bentonite as medium for the removal of Pb^2+^-containing waters or wastewaters

## 1 Data

**Fig. 1:**
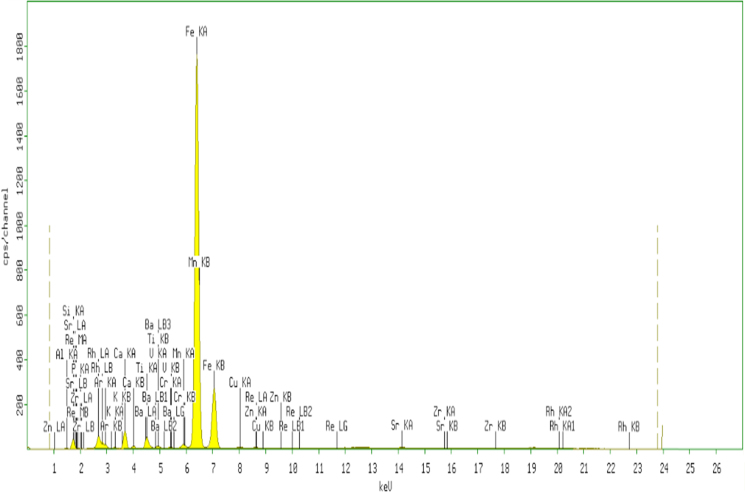
The XRF spectrum for the adsorbent from mixture of bagasse-bentonite before adsorption.

**Fig. 2:**
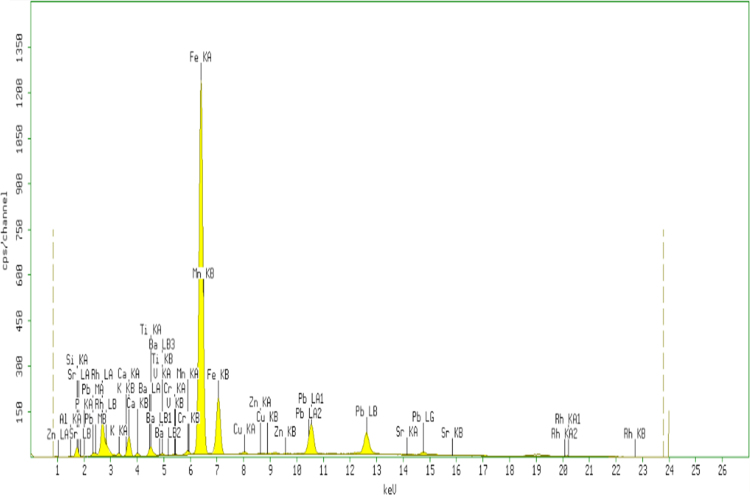
The XRF spectrum for the adsorbent from mixture of bagasse-bentonite after adsorption.

**Fig. 3:**
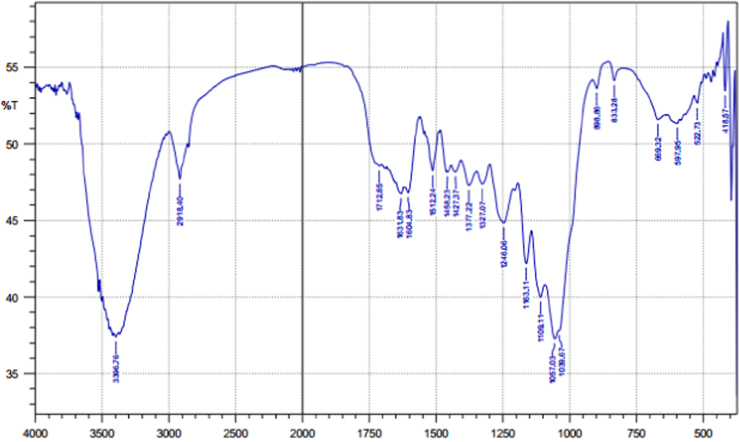
The FTIR spectrum for the adsorbent from mixture of bagasse-bentonite before adsorption.

**Fig. 4:**
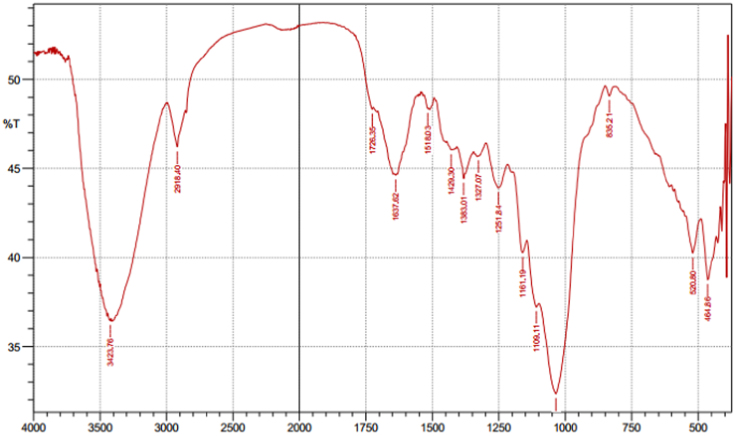
The FTIR spectrum for the adsorbent from mixture of bagasse-bentonite after adsorption.

**Fig. 5:**
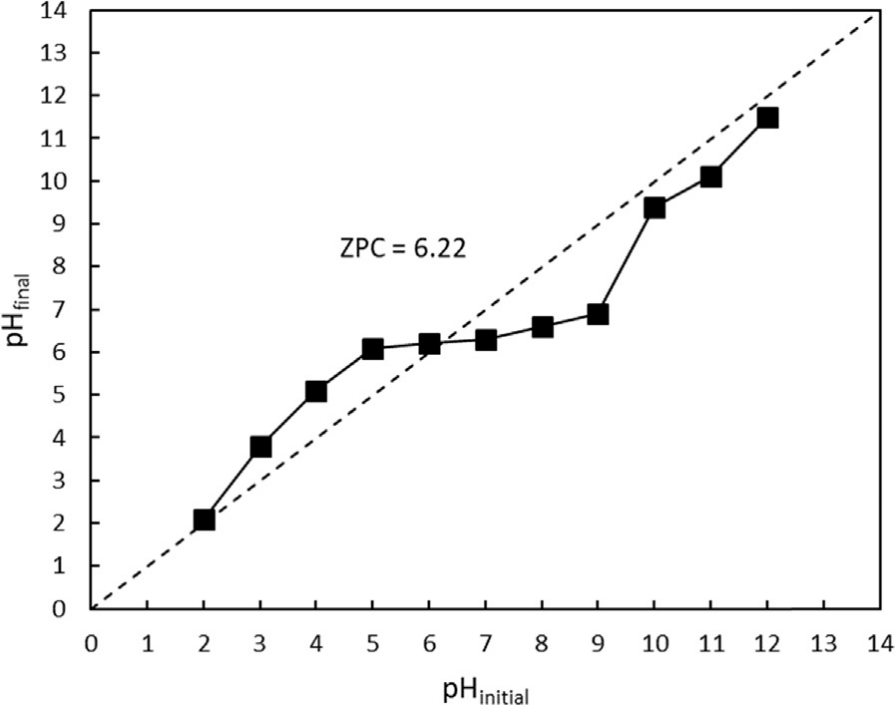
pH_final_ vs. pH_initial_ for mixture of bagasse-bentonite.

**Fig. 6:**
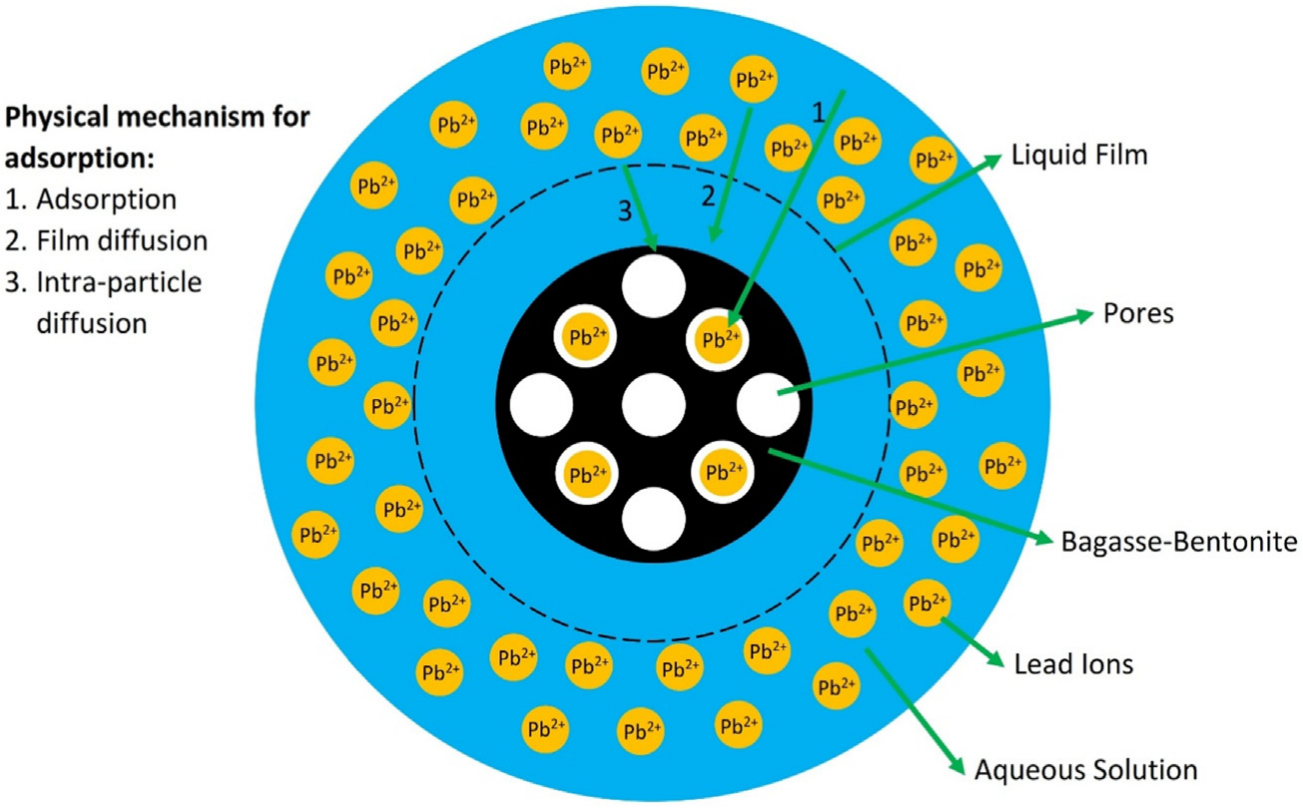
Schematic representation of physical mechanism for adsorption of Pb^2+^ onto mixture of bagasse-bentonite [2].

The XRF for the adsorbent from mixture of bagasse-bentonite before and after adsorption were given in Fig. 1, Fig. 2. The FTIR for the adsorbent from mixture of bagasse-bentonite before and after adsorption at wave numbers from 400 to 4000 cm^-1^ were given in Fig. 3, Fig. 4. The pH of zero point charge, pH_ZPC_, for mixture of bagasse-bentonite obtained is shown in Fig. 5. The adsorption of lead ions onto mixture of bagasse-bentonite is represented in Fig. 6. The optimum condition for Pb^2+^ adsorption on mixture of bagasse-bentonite is presented in Table 1. The kinetics and isotherms parameters were estimated using models listed in Table 2. The data of kinetics and isotherms for adsorption of lead ions onto the mixture of bagasse-bentonite is presented Table 3, Table 4.

**Table 1:** Optimum condition for Pb^2+^ adsorption on mixture of bagasse-bentonite (The concentration of Pb^2+^ solution is 100 mg/L).

Parameters	Optimum value	Adsorption efficiency (%)
Mixture ratio of bagasse and bentonite	3:1	97.31
pH	5	94.76
Time (min)	45	93.38
Average value		95.15

**Table 2:** Kinetic and Isotherm model/equations used in this data article [1], [2].

Model	Functional form	Plotting
Pseudo first order	$\ln\left(q_{e}-q_{t}\right)=\ln q_{e}-k_{1}t$	$\ln\left(q_{e}-q_{t}\right)$ vs *t*
Pseudo second order	$\frac{t}{q_{t}}=\frac{1}{k_{2}q_{e}^{2}}+\frac{t}{q_{e}}$	$\frac{t}{q_{t}}$ vs *t*
Intra-particle diffusion	$q_{t}=a+k_{\mathrm{int}}t^{0.5}$	*q*_*t*_ vs *t*^0.5^
Langmuir	$\frac{C_{e}}{q_{e}}=\frac{1}{q_{m}K_{L}}+\frac{1}{q_{m}}C_{e}$	$\frac{C_{e}}{q_{e}}$ vs $C_{e}$
Freundlich	$\log q_{e}=\log K_{f}+\frac{1}{n}\log C_{e}$	$\log q_{e}$ vs $\log C_{e}$

**Table 3:** Kinetics data for Pb^2+^ adsorbed onto the adsorbent from mixture of bagasse-bentonite.

Parameter	Value
Presudo first order	
*q_e_* (mg/g)	0.1434
*k*_1_ (min^-1^)	0.0149
*R*^2^	0.9717
Pseudo second order	
*q_e_* (mg/g)	9.4607
*k*_2_ (g/mg min)	0.4560
*R*^2^	0.9999
Intra-particle diffusion	
*C*	9.2955
*k_int_* (mg/g min^0.5^)	0.0128
*R*^2^	0.9649

**Table 4:** Isotherms data for Pb^2+^ adsorbed onto the adsorbent from mixture of bagasse-bentonite.

Parameter	Value
Langmuir	
*q_m_* (mg/g)	−4.2808
*K_L_* (L/mg)	0.0905
*R*^2^	0.8874
Freundlich	
*n*	0.5013
*K_f_* (L/g)	7.9524
*R*^2^	0.9005

*q_e_* and *q_t_* are amount of adsorbate which adsorbed (mg/g) at equilibrium and at *t* (min), *q_m_* is maximum adsorption capacity of the adsorbent (mg/g), *k*_1_ is the pseudo-first-order adsorption rate constant (min^-1^), *k*_2_ is the pseudo-second-order adsorption rate constant (g/mg min), *k_int_* is intra-particle diffusion rate constant (mg/g min^0.5^), *C_e_* is concentration of adsorbate in the solution at equilibrium (mg/L), *K_L_* is Langmuir constant (L/mg), *K_f_* is Freundlich constant (mg/g), and *n* is adsorption intensity.

## 2 Experimental design, materials and methods

### 2.1 Materials

The materials used in the present research are bagasse, bentonite, HCl 1 M, NaOH 1 M, aquademin, filter paper, label paper, Pb(NO_3_)_2_. The tools used in the present research is the crusher (mortar and pestle), beaker glass, 100 mL measuring cup, 10 mL volume pipette, stirring rod, 1000 mL measuring flask, 200 mesh strainer, pH meter, oven, bottle sample, analytical balance, Buchner funnel, shaker, suction pump, desiccator, and glass bottle.

### 2.2 Preparation of bagasse adsorbents

The bagasse taken from the sugar mill is cleaned by washing it with running water until it is cleaned and soaked in aquadest for 48 h, by changing the aquademin every 12 h. The bagasse was then dried under the sun and dried in an oven at 90 °C for 24 h, then crushed and sieved to 200 mesh [3].

### 2.3 Preparation of bentonite adsorbents

The first step in making bentonite adsorbents is to prepare 200 mesh bentonite. Bentonite is then heated in an oven at 105 °C for 24 h. This is performed to remove water content in bentonite [4].

### 2.4 Preparation of adsorbent from mixture of bagasse-bentonite

The first step of preparing the adsorbent of bagasse-bentonite is to mix bagasse and bentonite that have been weighed in accordance with the ratio of 3:1. Then the mixture is added with aquademin, stirred until the mixed adsorbent is completely mixed. After mixing, the mixture is heated in the oven at 105 °C for 24 h until the mixture is already dry. The dried mixture was then crushed and sieved with mesh 200 [5]. The mixture can further be used as an adsorbent.

### 2.5 Adsorption experiments

Adsorption of Pb^2+^ with the adsorbent of bagasse-bentonite was performed using batch adsorption technique. There are several experimental steps to determine the optimum condition of each variation. The shuffling of the sample was performed with a shaker at a speed of 150 rpm at room temperature. The water samples after shaking will be filtered using filter paper, then the sample water is tested with an Atomic Absorption Spectrophotometer (AAS) (repeated 3 times). The determination of adsorption kinetic type was performed by determining the adsorption capacity of Pb^2+^ solution on different time variations of 5, 10, 30, 45, 90, 120, and 180 min. The determination of adsorption isotherm type was performed by determining the adsorption capacity of Pb^2+^ solution on different concentration variations of 20, 40, 80, and 160 mg/L. Adsorbents are used according to the optimum mixture ratio of bagasse-bentonite (3:1), optimum pH (pH 5), and optimum contact time (45 min).

### 2.6 Characterization of adsorbent from mixture of bagasse-bentonite

The characterization of adsorbent from mixture of bagasse-bentonite for before and after adsorption was carried out using X-ray flourescence (XRF) and fourier transform infrared (FTIR). The characterization of adsorbent from mixture of bagasse-bentonite was carried out using X-ray flourescence (XRF) which aimed to analyze and to find out the elemental composition on the surface of the adsorbent samples and fourier transform infrared (FTIR) which aimed to analyze and to find out the functional groups of adsorbent from mixture of bagasse-bentonite.

### 2.7 pH_ZPC_ analysis

To determine pH_ZPC_ of the samples, 0.15 g adsorbent was added to 50 mL sodium chloride (NaCl; 0.01 mol L^−1^), and the solution pH was adjusted to the required pH in the range of 2–12 using hydrochloric acid (HCl; 0.1 mol L^−1^) and/or NaOH (0.1 mol L^−1^). Then, the mixture-containing Erlenmeyer flasks were agitated for 48 h at room temperature (28 °C) on a rotary shaker at 200 rpm.

## 3 Data analysis

The efficiency of Pb^2+^ adsorption by adsorbent from mixture of bagasse-bentonite is calculated according to Eq. (1).

(1) $$\mathrm{Efficiency adsorption}=\frac{C_{o}-C_{e}}{C_{o}}\cdot 100\%$$

where *C_o_* is initial concentration (mg/L) and *C_e_* is final concentration (mg/L).

While the adsorption capacity is calculated according to Eq. 2.

(2) $$q_{e}=\frac{V\cdot \left(C_{o}-C_{e}\right)}{m}$$

where *q_e_* is adsorption capacity per weight of the adsorbent (mg/g), *V* is volume of the solution (L), *C_o_* is initial concentration of solution (mg/L), *C_e_* is final concentration of solution (mg/L), *m* is mass of adsorbent (*g*).
